# Do Meta‐Analyses of Total Hip Arthroplasty Produce Reliable Results? A Systematic Review and Meta‐Epidemiological Study of Statistical Methods

**DOI:** 10.1111/os.70077

**Published:** 2025-05-27

**Authors:** Nikolai Ramadanov, Maximilian Voss, Radharani Michelle Diallo, Jonathan Lettner, Hassan Tarek Hakam, Robert Prill, Roland Becker, Robert Hable

**Affiliations:** ^1^ Center of Orthopaedics and Traumatology, Brandenburg Medical School University Hospital Brandenburg an der Havel Brandenburg an der Havel Germany; ^2^ Faculty of Health Science Brandenburg Brandenburg Medical School Theodor Fontane Brandenburg an der Havel Germany; ^3^ Faculty of Applied Computer Science Deggendorf Institute of Technology Deggendorf Germany

**Keywords:** heterogeneity, heterogeneity estimator, meta‐analyses, statistics

## Abstract

**Background:**

Total hip arthroplasty (THA) is a highly successful orthopedic procedure, with numerous meta‐analyses published to optimize its outcomes. However, the reliability of their results and conclusions depends heavily on the use of appropriate statistical methods. Therefore, the aim was to test the reliability of statistical methods in meta‐analyses of THA by examining the degree of heterogeneity, the effect of different between‐study variance estimators, and the equality of sample size of pooled primary studies.

**Methods:**

The literature was systematically searched in PubMed from January 1, 2022, to December 31, 2023, for meta‐analyses on THA. The quality of the meta‐analyses was assessed using the revised Measurement Tool to Assess Systematic Reviews (AMSTAR 2). All meta‐analyses were recalculated using eight different heterogeneity estimators. The following indicators were considered: inequality of patient numbers, proportion of random‐effects and fixed‐effects models, heterogeneity with *I* ^2^ value, ratio of effect sizes (RES), ratio of confidence interval width (RCIW), and the number of significant results. Mixed linear regression was then used to analyze whether the effect sizes and CIW were significantly different using different heterogeneity estimators. Finally, all examined meta‐analyses were recalculated using the eight heterogeneity estimators and the Hartung–Knapp (HK) adjustment.

**Results:**

Of the 24 meta‐analyses examined, 15 reported an outcome using a mean difference and 20 reported an outcome using an odds ratio. The quality assessment identified 10 meta‐analyses of high quality, 7 of moderate quality, 4 of low quality, and 3 of critically low quality. The significance of the examined meta‐analyses varied considerably depending on the heterogeneity estimators used. In particular, the DerSimonian and Laird and Hunter–Schmidt heterogeneity estimators tended to produce false‐positive results. The meta‐analyses examined generally did not use HK adjustment. This effect is amplified when combined with the weak DerSimonian and Laird heterogeneity estimator, which were used in almost all examined meta‐analyses.

**Conclusion:**

Without HK adjustment, the results depend strongly on the heterogeneity estimator chosen and there is a risk of false positives, especially for the widely used DerSimonian and Laird heterogeneity estimator. For HK adjustment, the choice of heterogeneity estimator seems to play a less important role. We recommend the use of more reliable heterogeneity estimators as well as the HK adjustment as a measure to improve the statistical methodology of meta‐analyses. This study highlights the critical need for improved statistical rigor in meta‐analyses of THA, ensuring more reliable evidence for clinical decision‐making and guideline development.

## Graphical abstract

Meta‐analyses of total hip arthroplasty (THA) were reevaluated using eight heterogeneity estimators to assess the risk of false positives. The Hartung–Knapp adjustment reduced estimator dependence, suggesting its use alongside more reliable estimators to improve statistical accuracy.

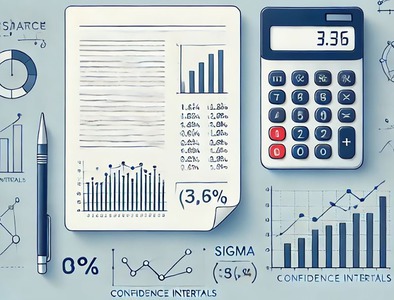

**Abbreviations**
AMSTAR: Measurement Tool to Assess Systematic Reviews
CI: confidence intervals
CIW: confidence intervals width
DL: DerSimonian and Laird
EB: empirical Bayes
HE: Hedges
HHS: Harris Hip score
HK: Hartung–Knapp
HS: Hunter–Schmidt
MCID: minimal clinically important difference
MD: mean difference
ML: maximum‐likelihood
OR: odds ratio
PM: Paule–Mandel
PROSPERO: International Prospective Register of Systematic Reviews
RCIW: ratio of confidence interval width
REML: restricted maximum likelihood
RES: ratio of effect sizes
SJ: Sidik–Jonkman
THA: total hip arthroplasty

## 1 Introduction

Total hip arthroplasty (THA) is widely recognized in the orthopedic scientific community as one of the most successful orthopedic procedures ever performed. Millions of patients with various hip pathologies have experienced relief from hip pain and a return to lost physical activities. In this context, it is essential that science provides us with reliable results to help us decide whether changes need to be made to established THA procedures. The best evidence on the effectiveness of treatments in clinical research comes from meta‐analyses. It is systematic reviews and meta‐analyses that form the top of the evidence‐based medicine pyramid [1] and are used to formulate guidelines. In recent years, dozens of meta‐analyses have been published on THA, attempting to optimize the outcome of THA through their conclusions. It is therefore extremely important to check whether these meta‐analyses produce statistically valid results.

A meta‐analysis is a statistical technique that combines the results of several trials on the same question, gathered through a systematic review of the literature, into meta‐results [2]. In order to obtain reliable combined meta‐results, the selection of an appropriate statistical model is of greatest importance for the validity of the meta‐analysis. In general, two main statistical models are used: the fixed‐effects model and the random‐effects model [3]. Basically, the main difference between the two is that the fixed‐effects model assumes that all the primary studies included in the meta‐analysis have the same true effect size, whereas the random‐effects model takes into account the variation in effect size between the pooled studies in the meta‐analysis. While the fixed‐effects model does not take into account heterogeneity between primary studies, the random‐effects model accounts for statistical heterogeneity between primary studies and assigns relatively more weight to smaller studies [4]. In the absence of heterogeneity, the random‐effects model would produce identical results to the fixed‐effects model. The random‐effects model uses different estimators of between‐study variance to calculate confidence intervals (CIs) [2]. The DerSimonian and Laird (DL) method is perhaps the most commonly used between‐study variance estimator [5]. DL is a relatively simple and weak method that can lead to false‐positive results. This is, particularly, the case in meta‐analyses with a small number of primary studies [6], when studies are unequally sized [7], or when heterogeneity is high [8]. To overcome the weakness of the DL estimator, many other alternatives have been introduced: the restricted maximum likelihood (REML) [9], the Paule–Mandel (PM) estimator [10], maximum‐likelihood (ML) [11], Hunter–Schmidt (HS) [12], the Sidik–Jonkman (SJ) [13] with or without Hartung–Knapp (HK) adjustment [14], Hedges (HE) [15], empirical Bayes (EB) [16] estimator.

The reliability of meta‐analyses depends on statistical methodology, particularly, in handling heterogeneity. Many THA meta‐analyses rely on the DL estimator, which is prone to false‐positive results, yet its impact in this field remains largely unexamined. Similarly, the HK adjustment, known to improve statistical robustness, is rarely applied. This study addresses these gaps by assessing how different heterogeneity estimators influence meta‐analysis results and evaluating the impact of HK adjustment.

The aim of this study was to test the reliability of statistical methods in meta‐analyses of THA by examining the degree of heterogeneity, the effect of different between‐study variance estimators, and the equality of sample sizes of pooled primary studies.

## 2 Methods

### 2.1 Study Registration and Search Strategy

The study protocol was registered in the International Prospective Register of Systematic Reviews (PROSPERO) on February 21, 2024 (CRD42024511637). The literature was systematically searched in PubMed from January 1, 2022, to December 31, 2023, using the following search terms: (total hip arthroplasty) AND (meta‐analysis). In a step‐by‐step screening process, records were first screened by title and abstract and then by full‐text analysis. The systematic search and screening process were carried out by two independent reviewers (N.R. and M.V.).

### 2.2 Eligibility Criteria

Inclusion criteria were as follows: any meta‐analysis comparing the Harris Hip score (HHS) of THA with the HHS of THA or any other type of intervention as well as any meta‐analysis comparing the complications of THA with the complications (or revision/reoperation) of THA or any other type of intervention. The HHS is probably the most widely used and important functional hip score. The HHS is a continuous outcome parameter, and the complications are a dichotomous outcome parameter often reported in meta‐analyses. Network meta‐analyses, meta‐analyses without information on HHS or complications, on mean difference (MD) or odds ratio (OR), and meta‐analyses with insufficient data were excluded. Meta‐analyses without forest plots were excluded because our data extraction process relied on extracting effect sizes, CIs, and heterogeneity estimates directly from the forest plots of the primary studies included in each meta‐analysis. Without these, a standardized and accurate extraction was not feasible.

### 2.3 Data Extraction and Quality Assessment

Data extraction was also carried out by two independent reviewers (M.V. and R.M.D.). Disagreements were again resolved by scientific discussion. The following characteristics of the meta‐analyses examined were extracted: journal title, year of publication, and country of origin; name of the first author of the meta‐analyses examined and of the primary studies; effect measure (MD, OR) with standard deviation and upper and lower CIs; statistical model used in the meta‐analyses examined (random‐effects or fixed‐effects model); level of statistical heterogeneity; number of primary studies included in the meta‐analyses; number of participants; between‐study variance estimator used in the meta‐analyses; information on the outcome parameter examined in the meta‐analyses examined. Some meta‐analyses presented data on HHS at different time intervals after surgery and data on different types of complications. In these cases, we restricted ourselves to these outcomes, which were calculated based on the largest number of primary trials, to ensure the clarity of our analysis. The quality of the meta‐analyses examined was assessed using the revised Measurement Tool to Assess Systematic Reviews (AMSTAR 2) [17].

### 2.4 Selection of Heterogeneity Estimators

Eight heterogeneity estimators were selected based on their widespread use in meta‐analyses and their statistical properties. DL [5] is the most commonly applied estimator but has been criticized for producing false‐positive results in cases of high heterogeneity. Alternatives such as REML [9], PM [10], ML [11], and EB [16] offer more robust variance estimation. HS [12], HE [15], and SJ [13] were included for their distinct methodological approaches, with SJ being, particularly, relevant due to its compatibility with the HK adjustment. These methods allow a comprehensive evaluation of the impact of heterogeneity estimation on meta‐analytic results.

### 2.5 Statistical Analysis

#### 2.5.1 Descriptive Analysis

First, the results of all meta‐analyses examined were recalculated using the different heterogeneity estimators (DL, REML, PM, ML, HS, SJ, HE, EB) based on their primary studies. In the descriptive part of the analysis, the average number of primary studies per meta‐analysis examined, the inequality of patient numbers in the primary studies, the proportion of random‐effects and fixed‐effects models, the heterogeneity with *I* ^2^ value, the ratio of effect sizes (RES), the ratio of confidence interval width (RCIW), and the number of significant results were calculated for each heterogeneity estimator.

The indicator ‘inequality of patient numbers’ measures the extent to which the primary studies in a meta‐analysis differ in the number of patients included. For this purpose, the following was calculated for each meta‐analysis (*i*) examined: $\text{Inequality of patient numbers}\;\left(i\right)=\frac{\text{Range}\;\left(N_{i,1},N_{i,2},\ldots ,N_{i,m}\right)}{\text{Median}\;\left(N_{i,1},N_{i,2},\ldots ,N_{i,m}\right)}\times 100\%$, where $N_{i,j}$ is the number of patients in primary study (*j*) in meta‐analysis examined (*i*). This measure represents the range of patient numbers (maximum—minimum) in relation to the median patient number. A value below 200% was defined as “moderate inequality,” a value between 200% and 1000% as “high inequality,” and a value above 1000% as “excessive inequality.” Groups were also created for the *I* ^2^ value, with an *I* ^2^ value below 30% indicating low heterogeneity, an *I* ^2^ value between 30% and 60% indicating moderate heterogeneity, and an *I* ^2^ value above 60% indicating substantial heterogeneity. The indicator RES compares the extent to which the effect size from the meta‐analysis examined changes when a different heterogeneity estimator is used in the meta‐analysis calculation. Effect size refers to the absolute measure of the observed effect in the meta‐analysis (e.g., MD or OR). In contrast, the RES is a comparative measure used to assess how the choice of heterogeneity estimator influences effect size calculations. The indicator RES compares the extent to which the effect size from the meta‐analysis examined changes when a different heterogeneity estimator is used in the meta‐analysis calculation. The comparison value in each case is the effect size when using DL as the standard heterogeneity estimator. If ${\mathrm{ES}}_{i,\text{REML}}$ denotes the effect size obtained when using the REML heterogeneity estimator in the meta‐analysis examined *i*, for example, then the RES for this is equal to ${\mathrm{RES}}_{i,\text{REML}}=\frac{{\mathrm{ES}}_{i,\text{REML}}}{{\mathrm{ES}}_{i,\mathrm{DL}}}$. The indicator RCIW, similar to RES, compares how the width of the confidence interval (CIW) changes when a different heterogeneity estimator is used instead of the DL heterogeneity estimator, for example, the REML estimator: ${\text{RCIW}}_{i,\text{REML}}=\frac{{\mathrm{CIW}}_{i,\text{REML}}}{{\mathrm{CIW}}_{i,\mathrm{DL}}}$. The indicator “number of significant results” shows the number of meta‐analyses examined with significant results depending on the heterogeneity estimator used.

These results were then systematically and statistically analyzed separately for MD and OR meta‐analysis to determine the extent to which the meta‐analysis results differed according to the heterogeneity estimator.

#### 2.5.2 Effect Size Comparison

Mixed linear regression was used to analyze whether the effect sizes using alternative heterogeneity estimators were significantly different from the effect sizes using the DL estimator. For this purpose, the following mixed model was calculated with RES as a dependent variable, the respective heterogeneity estimator as a fixed effect and the respective meta‐analysis as a random effect: ${\mathrm{RES}}_{i,h}=1+\beta_{h}+\tau_{i}+\varepsilon_{i,h}$ for meta‐analyses i=1,…,N and heterogeneity estimators h = REML, PM, ML, HS, SJ, HE, EB where $\beta_{h}$ denotes the fixed effect, $\tau_{i}$ the random effect and $\varepsilon_{i,h}$ the error term. As by definition ${\mathrm{RES}}_{i,\mathrm{DL}}=\frac{{\mathrm{ES}}_{i,\mathrm{DL}}}{{\mathrm{ES}}_{i,\mathrm{DL}}}=1$, the intercept is fixed equal to 1.

#### 2.5.3 Confidence Intervals Comparison

Mixed linear regression was used to analyze whether the CIW when using alternative heterogeneity estimators differs significantly from the CIW when using the DL estimator. For this purpose, the following mixed model was calculated with RCIW as a dependent variable, the respective heterogeneity estimator as a fixed effect, and the respective meta‐analysis as a random effect: ${\text{RCWI}}_{i,h}=1+\beta_{h}+\tau_{i}+\varepsilon_{i,h}$ for meta‐analyses i=1,…,N and heterogeneity estimators h = REML, PM, ML, HS, SJ, HE, EB where $\beta_{h}$ denotes the fixed effect, $\tau_{i}$ the random effect and $\varepsilon_{i,h}$ the error term. As by definition ${\text{RCWI}}_{i,\mathrm{DL}}=\frac{{\mathrm{CWI}}_{i,\mathrm{DL}}}{{\mathrm{CWI}}_{i,\mathrm{DL}}}=1$, the intercept is fixed equal to 1 again.

#### 2.5.4 Influence of Study Characteristics

The extent to which other influencing variables have an impact on the difference in effect size between each heterogeneity estimation method and the DL estimator was calculated separately for each method. A linear regression was calculated for each heterogeneity estimator (DL, REML, PM, ML, HS, SJ, HE, EB) with a dependent variable: RES. The independent variables were chosen based on their known influence on meta‐analytic effect sizes: (1) number of studies per meta‐analysis, as smaller studies tend to yield more variable estimates; (2) inequality of patient numbers, since large disparities in sample sizes can affect weighting and significance; and (3) *I* ^2^ (< 30% or ≥ 30%), as high heterogeneity may alter the impact of different estimators. In line with the above analysis, the extent to which other influencing variables have an impact on the difference in CIW between each heterogeneity estimation method and the DL estimator was calculated separately for each method. For this purpose, a linear regression was calculated for each method (DL, REML, PM, ML, HS, SJ, HE, EB) with a dependent variable: RCIW; and independent variables: number of primary studies per meta‐analysis, inequality of patient numbers, and *I* ^2^ (< 30% or ≥ 30%).

#### 2.5.5 HK Adjustment

Finally, the entire calculation of the meta‐analyses examined with the 8 heterogeneity estimators was recalculated using the HK adjustment. All statistical analyses were performed by a professional statistician (RH) using the netmeta and metaphor packages in the R software version 4.2.1 (R Foundation for Statistical Computing, Vienna, Austria) [18].

## 3 Results

### 3.1 Systematic Literature Search

**FIGURE 1:**
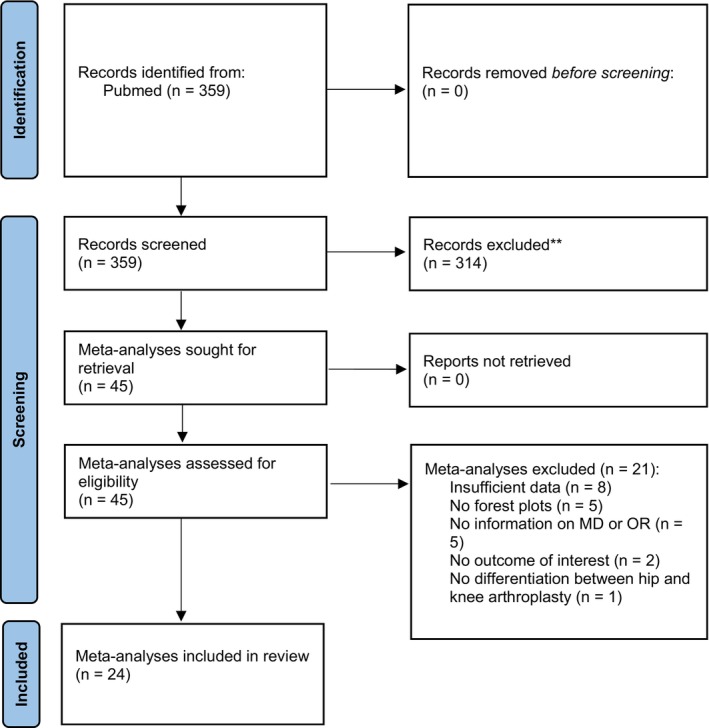
PRISMA flow diagram. PRISMA: Preferred Reporting Items for Systematic Reviews and Meta‐Analyses; MD: mean difference; OR: odds ration.

The initial literature search identified 359 records in PubMed from January 1, 2022, to December 31, 2023 (Figure 1). Of these 359 records, 45 meta‐analyses were screened by full‐text analysis [19, 20, 21, 22, 23, 24, 25, 26, 27, 28, 29, 30, 31, 32, 33, 34, 35, 36, 37, 38, 39, 40, 41, 42, 43, 44, 45, 46, 47, 48, 49, 50, 51, 52, 53, 54, 55, 56, 57, 58, 59, 60, 61, 62, 63]. Of these 45 meta‐analyses, 21 [43, 44, 45, 46, 47, 48, 49, 50, 51, 52, 53, 54, 55, 56, 57, 58, 59, 60, 61, 62, 63] were excluded for the following reasons: 8 meta‐analyses had insufficient data [43, 44, 45, 46, 47, 48, 49, 50], 5 meta‐analyses did not provide information on MD or OR [51, 52, 53, 54, 55], 5 meta‐analyses had no forest plots [56, 57, 58, 59, 60], 2 meta‐analyses had no outcome of interest [61, 62], and 1 meta‐analysis did not differentiate between hip and knee arthroplasty [63]. Finally, 24 meta‐analyses were considered eligible for analysis [19, 20, 21, 22, 23, 24, 25, 26, 27, 28, 29, 30, 31, 32, 33, 34, 35, 36, 37, 38, 39, 40, 41, 42]. The main characteristics of the meta‐analyses examined are presented in Table 1.

**TABLE 1:** Characteristics of the meta‐analyses examined [15, 16, 17, 18, 19, 20, 21, 22, 23, 24, 25, 26, 27, 28, 29, 30, 31, 32, 33, 34, 35, 36, 37].

Author	Publication year	Origin	Number of including studies in meta‐analyses	Number of including patients in meta‐analyses	Experimental group	Control group	Extracted outcome parameter	Effect measure	Heterogeneity variance estimator	Variance components model
Acuna et al. [19]	2022	USA	28	653,633	DAA THA	THA through other approach	Infection	OR	DL	REM
Ang et al. [20]	2023	Australia	24	2010	DAA THA	THA through other approach	HHS	MD	DL	REM
							Periprosthetic fracture	OR		REM
Arakawa et al. [21]	2023	Japan	15	13,159	THA with prior arthroscopy	THA without prior arthroscopy	HHS	MD	DL	FEM
							Overall complications	OR		FEM
Avila et al. [22]	2022	USA	11	278,782	THA with prior intraarticular injection	THA without prior intraarticular injection	Infection	OR	DL	REM
Awad et al. [23]	2023	USA	30	11,562	DAA THA	PA THA	Overall complications	OR	DL	REM
Chang et al. [24]	2023	South Korea	10	3716	THA through anterior‐based muscle‐sparing	DAA THA	Dislocation	OR	DL	REM
Chen et al. [25]	2023	China	22	1923	Bone marrow mononuclear cells before THA	Core decompression only and core decompression combined before THA	HHS	MD	DL	REM
							Overall complication	OR		FEM
Dockery et al. [26]	2023	USA	5	24,407	DAA THA	THA through other approach	Infection	OR	DL	FEM
Hoskins et al. [27]	2022	Australia/UK	9	8990	Dual mobility THA	Large femoral head bearing THA	Revision	OR	DL	FEM
Huang et al. [28]	2022	China	10	29,918	THA in systemic lupus erythematosus	THA in non‐ systemic lupus erythematosus	HHS	MD	DL	FEM
							Infection	OR		FEM
Kobayashi et al. [29]	2023	Japan	12	5525	THA with capsular repair	THA with capsulotomy	HHS	MD	DL	REM
							Dislocation	OR		REM
Kumar et al. [30]	2022	India	6	612	Hip resurfacing arthroplasty	THA	HHS	MD	DL	FEM
							Overall complications	OR		FEM
Kumar et al. [31]	2023	India	17	3600	Robot‐assisted THA	Conventional THA	HHS	MD	DL	REM
							Overall complications	OR		REM
Miura et al. [32]	2022	Japan	4	200	Acetabular cup fixation with screws in THA	Acetabular cup fixation with screw in THA	HHS	MD	DL	REM
Ramadanov [33]	2022	Germany	14	1021	SuperPATH THA	Conventional THA	HHS	MD	DL	REM
							Overall complications	OR		REM
Ramadanov et al. [34]	2022	Germany/Spain	20	1501	SuperPATH THA	Conventional THA	HHS	MD	DL	REM
Ramezani et al. [35]	2022	Iran	38	104,151	Simultaneous bilateral THA	Staged bilateral THA	Overall complications	OR	DL	FEM
Salman et al. [36]	2023	Qatar/UK/Jordan	14	211,102	THA in patients with osteonecrosis	THA in patients with osteoarthritis	HHS	MD	PM	REM
							Revision	OR		REM
Shigemura t al. [37]	2021	Japan	6	495	Anterolateral approach THA	Lateral approach THA	HHS	MD	DL	FEM
Tan et al. [38]	2022	Australia	67	8335	THA with intra‐articular injection of tranexamic acid	THA with intravenous administration of tranexamic acid	DVT	OR	DL	FEM
Wang et al. [39]	2023	China	10	2188	Revision THA with modular stems	Revision THA with monoblock stems	HHS	MD	DL	FEM
							Dislocation	OR		FEM
Wang et al. [40]	2022	China	18	2845	Robot‐assisted THA	Conventional THA	HHS	MD	DL	REM
							Overall complications	OR		FEM
Yang et al. [41]	2023	China	9	1,822,198	THA in obese patients	THA in non‐obese patients	Infection	OR	DL	FEM
Zhang et al. [42]	2023	China	7	4306	Direct superior approach THA	Conventional THA	HHS	MD	DL	FEM

Abbreviations: DAA: direct anterior approach; DL: DerSimonian and Laird estimator; DVT: deep vein thrombosis; FEM: fixed effects model; HHS: Harris Hip score; MD: mean difference; OR: odds ratio; PM: Paule–Mandel; REM: random effects model; THA: total hip arthroplasty.

### 3.2 Quality Assessment

The quality assessment of the meta‐analyses examined resulted in 10 meta‐analyses of high quality [19, 25, 26, 28, 29, 32, 33, 36, 38, 41], 7 meta‐analyses of moderate quality [21, 30, 31, 35, 37, 39, 42], 4 meta‐analyses of low quality [20, 24, 27, 34], and 3 meta‐analyses of critically low quality [22, 23, 40] (Table 2).

**TABLE 2:** Quality assessment using AMSTAR 2.

Author	Protocol registered before commencement of the review	Adequacy of the literature search	Justification for excluding individual studies	Risk of bias from individual studies being included in the review	Appropriateness of meta‐analytical methods	Consideration of risk of bias when interpreting the results of the review	Assessment of presence and likely impact of publication bias	Overall quality
Acuna et al. [19]	No	Yes	Yes	Yes	Yes	Partially yes	Yes	High
Ang et al. [20]	No	Yes	Yes	Yes	Yes	No	No	Low
Arakawa et al. [21]	Yes	Partially yes	No	Yes	Yes	Partially yes	Yes	Moderate
Avila et al. [22]	No	Yes	Partially yes	Yes	Yes	No	No	Critically low
Awad et al. [23]	Partially yes	Yes	Yes	No	Yes	No	No	Critically low
Chang et al. [24]	No	Yes	Yes	Yes	Yes	No	No	Low
Chen et al. [25]	Partially yes	Yes	Yes	Yes	Yes	No	Yes	High
Dockery et al. [26]	Yes	Yes	Yes	Yes	Yes	Yes	Yes	High
Hoskins et al. [27]	Partially yes	Yes	Partially yes	Yes	Yes	No	No	Low
Huang et al. [28]	No	Yes	Yes	Yes	Yes	Yes	Yes	High
Kobayashi et al. [29]	Yes	Yes	Yes	Yes	Yes	Yes	Yes	High
Kumar et al. [30]	No	Yes	Yes	Yes	Yes	Yes	No	Moderate
Kumar et al. [31]	Partially yes	Yes	Yes	Yes	Yes	Partially yes	No	Moderate
Miura et al. [32]	Yes	Yes	Yes	Yes	Yes	Yes	No	High
Ramadanov [33]	Yes	Yes	Partially yes	Yes	Yes	Yes	Yes	High
Ramadanov et al. [34]	Yes	Yes	Partially yes	Yes	Yes	No	No	Low
Ramezani et al. [35]	Yes	Yes	Yes	Yes	Partially yes	No	Yes	Moderate
Salman et al. [36]	Yes	Yes	Partially yes	Yes	Yes	Yes	Yes	High
Shigemura et al. [37]	No	Yes	Yes	Yes	Yes	No	Yes	Moderate
Tan et al. [38]	Yes	Yes	Yes	Yes	Yes	Yes	Yes	High
Wang et al. [39]	Yes	Yes	Yes	Yes	Yes	No	No	Moderate
Wang et al. [40]	No	Yes	Partially yes	Partially yes	No	Partially yes	No	Critically low
Yang et al. [41]	No	Yes	Yes	Yes	Yes	Partially yes	Yes	High
Zhang et al. [42]	Yes	Yes	Yes	Yes	Yes	No	No	Moderate

Abbreviation: AMSTAR: Measurement Tool to Assess Systematic Reviews.

### 3.3 Statistical Analysis

#### 3.3.1 Descriptive Overview of the Meta‐Analyses Examined

Table 3 gives a descriptive overview of the eight different meta‐analysis results, using the eight different heterogeneity estimators (DL, REML, PM, ML, HS, SJ, HE, EB). Of the meta‐analyses examined, 15 meta‐analyses reported the outcome of interest using MD, and 20 meta‐analyses reported the outcome of interest using OR. The average number of primary studies included in the meta‐analyses examined was 5.8, ranging from 2 to 11 for MD, and 10.8, ranging from 5 to 28 for OR. Table 3 shows that 45% of OR meta‐analyses had excessive inequality of patient numbers in the primary studies. A random effects model was used in 60% of the MD meta‐analyses and 45% of the OR meta‐analyses. For MD, 40% of meta‐analyses examined show substantial heterogeneity, and for OR, 25% of meta‐analyses examined show substantial heterogeneity (*I* ^2^ > 60%). Table 3 shows the average of RES across all meta‐analyses examined, with the range (minimum and maximum) in parentheses. For example, in the MD meta‐analyses, the effect sizes for REML do not differ on average from the effect sizes for DL, as the average RES value is 1. However, there are considerable differences in individual cases, as the minimum RES value for REML is 0.5. This means that in one of the meta‐analyses examined, the effect size obtained using REML is only half as large (RES = 0.5) as that obtained using DL. Table 3 shows the average RCIW across all meta‐analyses examined, with the range (minimum and maximum) in parentheses. For example, the use of the SJ heterogeneity estimator leads to wider CIs than the use of the DL heterogeneity estimator. In the MD meta‐analyses, the CIs are on average 40% wider (average RCIW for SJ: 1.4) and in the OR meta‐analyses, the CIs are on average 80% wider (average RCIW for SJ: 1.8). The indicator “number of significant results” showed considerable differences in MD meta‐analysis: using the standard DL heterogeneity estimator or the HS estimator, the results of five studies are significant, whereas using the SJ and HE estimators, only two studies would be significant. This means that 60% of the results obtained with DL become non‐significant when SJ is used. In OR meta‐analysis, the differences were smaller. Out of 20 OR outcomes, seven to nine were significant when using the 8 different heterogeneity estimators.

**TABLE 3:** Descriptive overview of the meta‐analyses examined.

Parameter	MD	OR
Number of meta‐analyses, *n*	15	20
Number of studies per meta‐analysis, mean value (min.–max.)	5.8 (2–11)	10.8 (5–28)
Inequality of patient numbers, *n*	Moderate: 12 (80%)	Moderate: 8 (40%)
	High: 3 (20%)	High: 3 (15%)
	Excessive: 0 (0%)	Excessive: 9 (45%)
Model, *n* (%)	Fixed effects: 6 (40%)	Fixed effects: 11 (55%)
	Random effects: 9 (60%)	Random effects: 9 (45%)
*I* ^2^, *n* (%)	< 30: 7 (46.7%)	< 30: 9 (45%)
	30–60: 2 (13.3%)	30–60: 6 (30%)
	> 60: 6 (40%)	> 60: 5 (25%)
*τ* ^2^, mean value (min.–max.)	DL: 4 (0–25.9)	DL: 0.3 (0–2)
	REML: 4.9 (0–20.5)	REML: 0.3 (0–2.2)
	PM: 5.6 (0–22.5)	PM: 0.3 (0–2.1)
	ML: 3.6 (0–14.3)	ML: 0.2 (0–1.4)
	HS: 2.3 (0–14.1)	HS: 0.2 (0–1.2)
	SJ: 6.8 (0–23.2)	SJ: 0.6 (0–2.3)
	HE: 5.9 (0–27.2)	HE: 0.3 (0–1.9)
	EB: 5.6 (0–22.5)	EB: 0.3 (0–2.1)
Ratio of effect sizes (RES), mean value (min.–max.)	REML: 1 (0.5–1.4)	REML: 1 (0.9–1.1)
	PM: 1 (0.9–1.5)	PM: 1 (1–1.1)
	ML: 1.2 (0.8–3.7)	ML: 1 (0.9–1.1)
	HS: 1 (0.6–1.5)	HS: 1 (0.9–1.1)
	SJ: 1.1 (0.2–2)	SJ: 1 (0.8–1.2)
	HE: 0.9 (−0.3–1.8)	HE: 1 (0.8–1.2)
	EB: 1 (0.9–1.5)	EB: 1 (1–1.1)
Ratio of CI width (RCIW), mean value (min.–max.)	REML: 1.1 (0.9–1.4)	REML: 1.1 (0.8–1.8)
	PM: 1.1 (0.8–1.6)	PM: 1.1 (0.8–2)
	ML: 1 (0.6–1.3)	ML: 1 (0.6–1.5)
	HS: 0.9 (0.7–1)	HS: 0.9 (0.7–1)
	SJ: 1.4 (0.9–2.3)	SJ: 1.8 (1–4.5)
	HE: 1.1 (0.3–2)	HE: 1.1 (0.4–3.8)
	EB: 1.1 (0.8–1.6)	EB: 1.1 (0.8–2)
Number of significant results, *n* (%)	DL: 5 (33.3%)	DL: 8 (40%)
	REML: 3 (20%)	REML: 8 (40%)
	PM: 3 (20%)	PM: 7 (35%)
	ML: 3 (20%)	ML: 8 (40%)
	HS: 5 (33.3%)	HS: 8 (40%)
	SJ: 2 (13.3%)	SJ: 7 (35%)
	HE: 2 (13.3%)	HE: 9 (45%)
	EB: 3 (20%)	EB: 7 (35%)

Abbreviations: DL: DerSimonian–Laird estimator; EB: empirical Bayes estimator; HE: Hedges estimator; HS: Hunter–Schmidt estimator; MD: mean difference; ML: maximum‐likelihood estimator; OR: odds ratio; PM: Paule–Mandel estimator; REML: restricted maximum‐likelihood estimator; SJ: Hartung–Knapp–Sidik–Jonkman estimator.

#### 3.3.2 Test for Differences in Effect Size

In the MD meta‐analyses, the effect sizes obtained with the other heterogeneity estimators were not significantly different from the effect sizes obtained with the DL estimator (Table 4). In the OR meta‐analyses, only the effect sizes obtained with the SJ estimator differed significantly (*p* = 0.0134) from those obtained with the DL estimator (Table 4). For the SJ estimator, the effect sizes are slightly higher by 3% (coefficient = 0.03).

**TABLE 4:** Test for differences in effect size.

Method	Coefficient	Standard error	*t*	*p*
MD				
EB	0.02	0.10	0.18	0.8558
HE	−0.10	0.10	−0.98	0.3299
HS	−0.00	0.10	−0.04	0.9653
ML	0.15	0.10	1.54	0.1268
PM	0.02	0.10	0.18	0.8558
REML	−0.00	0.10	−0.03	0.9763
SJ	0.11	0.10	1.08	0.2839
OR				
EB	0.01	0.01	0.77	0.4409
HE	−0.01	0.01	−0.45	0.6556
HS	−0.01	0.01	−1.17	0.2427
ML	0.00	0.01	0.37	0.7113
PM	0.01	0.01	0.77	0.4408
REML	0.01	0.01	0.88	0.3819
SJ	0.03	0.01	2.49	0.0143*

Abbreviations: EB: empirical Bayes estimator; HE: Hedges estimator; HS: Hunter–Schmidt estimator; MD: mean difference; ML: maximum‐likelihood estimator; OR: odds ratio; PM: Paule–Mandel estimator; REML: restricted maximum‐likelihood estimator; SJ: Hartung–Knapp–Sidik–Jonkman estimator.

* Significant results.

#### 3.3.3 Test for Differences in the Width of Confidence Intervals

In the MD meta‐analyses, only the CIW calculated with the SJ estimator differs significantly (*p* < 0.0001) from those obtained with the DL estimator (Table 5). However, the difference is substantial: with SJ, the CIs are 35% (coefficient 0.35) wider than with the DL estimator. In the OR meta‐analyses, only the CIW calculated with the SJ estimator differs significantly (*p* < 0.0001) from those obtained with the DL estimator (Table 5). The difference is even greater than in the MD meta‐analyses: with SJ, the CIs are 79% (coefficient 0.79) wider than with the DL estimator.

**TABLE 5:** Test for differences in the width of confidence intervals.

Method	Coefficient	Standard error	t value	P value
MD				
EB	0.08	0.07	1.08	0.2871
HE	0.09	0.07	1.28	0.2052
HS	−0.10	0.07	−1.42	0.1633
ML	−0.04	0.07	−0.52	0.6035
PM	0.08	0.07	1.08	0.2871
REML	0.09	0.07	1.17	0.2497
SJ	0.35	0.07	4.81	< 0.0001***
OR				
EB	0.12	0.12	1.05	0.2971
HE	0.11	0.12	0.96	0.3408
HS	−0.11	0.12	−0.92	0.3610
ML	−0.04	0.12	−0.32	0.7521
PM	0.12	0.12	1.05	0.2970
REML	0.10	0.12	0.80	0.4226
SJ	0.79	0.12	6.64	< 0.0001***

Abbreviations: EB: empirical Bayes estimator; HE: Hedges estimator; HS: Hunter–Schmidt estimator; MD: mean difference; ML: maximum‐likelihood estimator; OR: odds ratio; PM: Paule–Mandel estimator; REML: restricted maximum‐likelihood estimator; SJ: Hartung–Knapp–Sidik–Jonkman estimator.

*** Highly significant results.

#### 3.3.4 Other Factors Influencing the Change in Effect Size

For MD and OR meta‐analyses, the influence of other factors on the change in effect size was examined using linear regression for each heterogeneity estimator (Table 6). As an example of how to interpret the results in Table 6, the following text describes the results for the MD meta‐analyses using the PM estimator. The number of primary studies used in the meta‐analysis (*p* = 0.0323) and the inequality of patient numbers (*p* < 0.0001) have a significant effects. For each additional primary study, the effect size using PM decreases by approximately 1.9% compared to the DL estimator (coefficient = −0.0192). This means that as the number of studies increases, the effect size obtained with the PM estimator is smaller than that obtained with the DL estimator. The more unequal the number of patients in the primary studies, the higher the effect size obtained with PM compared to DL.

**TABLE 6:** Other factors influencing the change in effect size.

MD				
Parameter	Coefficient	Standard error	*t*	*p*
REML				
(Intercept)	0.8865	0.1152	7.6957	< 0.0001***
Number of studies	0.0015	0.0190	0.0786	0.9388
Unequality of number of patients	0.0006	0.0003	2.0886	0.0608
*I* ^2^ ≥ 30%	−0.0005	0.1076	−0.0046	0.9964
PM				
(Intercept)	1.0078	0.0476	21.1696	< 0.0001***
Number of studies	−0.0192	0.0079	−2.4492	0.0323*
Unequality of number of patients	0.0007	0.0001	6.2507	< 0.0001***
*I* ^2^ ≥ 30%	−0.0084	0.0445	−0.1889	0.8536
ML				
(Intercept)	0.9614	0.4744	2.0265	0.0677
Number of studies	0.0264	0.0783	0.3375	0.7421
Unequality of number of patients	−0.0010	0.0012	−0.8553	0.4106
*I* ^2^ ≥ 30%	0.3937	0.4433	0.8881	0.3935
HS				
(Intercept)	1.0774	0.1024	10.5182	< 0.0001***
Number of studies	−0.0128	0.0169	−0.7569	0.4650
Unequality of number of patients	−0.0007	0.0003	−2.7637	0.0184*
*I* ^2^ ≥ 30%	0.2112	0.0957	2.2070	0.0495*
SJ				
(Intercept)	0.7535	0.2908	2.5912	0.0251*
Number of studies	0.0283	0.0480	0.5893	0.5676
Unequality of number of patients	0.0010	0.0007	1.4186	0.1837
*I* ^2^ ≥ 30%	0.0261	0.2717	0.0960	0.9252
HE				
(Intercept)	1.2890	0.1910	6.7493	< 0.0001***
Number of studies	−0.0441	0.0315	−1.3979	0.1897
Unequality of number of patients	−0.0021	0.0005	−4.4420	0.0010***
*I* ^2^ ≥ 30%	0.4310	0.1784	2.4152	0.0343*
EB				
(Intercept)	1.0078	0.0476	21.1696	< 0.0001***
Number of studies	−0.0192	0.0079	−2.4492	0.0323*
Unequality of number of patients	0.0007	0.0001	6.2507	< 0.0001***
*I* ^2^ ≥ 30%	−0.0084	0.0445	−0.1889	0.8536

OR				
Parameter	Coefficient	Standard error	*t*	*p*
REML				
(Intercept)	1.0007	0.0154	64.7680	< 0.0001***
Number of studies	−0.0006	0.0012	−0.4752	0.6411
Unequality of number of patients	0.0000	0.0000	0.4558	0.6547
*I* ^2^ ≥ 30%	0.0266	0.0147	1.8106	0.0890
PM				
(Intercept)	1.0070	0.0138	72.7933	< 0.0001***
Number of studies	−0.0008	0.0011	−0.7536	0.462
Unequality of number of patients	−0.0000	0.0000	−0.6581	0.5198
*I* ^2^ ≥ 30%	0.0222	0.0132	1.6903	0.1104
ML				
(Intercept)	1.0009	0.0193	51.9088	< 0.0001***
Number of studies	−0.0002	0.0015	−0.1045	0.9181
Unequality of number of patients	−0.0000	0.0000	−0.4664	0.6472
*I* ^2^ ≥ 30%	0.0118	0.0183	0.6420	0.53
HS				
(Intercept)	0.9932	0.0214	46.4117	< 0.0001***
Number of studies	0.0004	0.0017	0.2527	0.8037
Unequality of number of patients	−0.0000	0.0000	−0.5696	0.5769
*I* ^2^ ≥ 30%	−0.0172	0.0204	−0.8442	0.411
SJ				
(Intercept)	0.9827	0.0365	26.9163	< 0.0001***
Number of studies	−0.0004	0.0029	−0.1234	0.9033
Unequality of number of patients	0.0000	0.0000	2.1187	0.0501
*I* ^2^ ≥ 30%	0.0687	0.0347	1.9789	0.0653
HE				
(Intercept)	1.0463	0.0380	27.5475	< 0.0001***
Number of studies	−0.0058	0.0030	−1.8994	0.0757
Unequality of number of patients	−0.0000	0.0000	−0.5496	0.5902
*I* ^2^ ≥ 30%	0.0253	0.0361	0.6996	0.4942
EB				
(Intercept)	1.0070	0.0138	72.7919	< 0.0001***
Number of studies	−0.0008	0.0011	−0.7536	0.462
Unequality of number of patients	−0.0000	0.0000	−0.6590	0.5193
*I* ^2^ ≥ 30%	0.0223	0.0132	1.6905	0.1103

Abbreviations: EB: empirical Bayes estimator; HE: Hedges estimator; HS: Hunter–Schmidt estimator; MD: mean difference; ML: maximum‐likelihood estimator; OR: odds ratio; PM: Paule–Mandel estimator; REML: restricted maximum‐likelihood estimator; SJ: Hartung–Knapp–Sidik–Jonkman estimator.

* Significant results.

*** Highly significant results.

#### 3.3.5 Other Factors Influencing the Change in Confidence Interval Width

For MD and OR meta‐analyses, the influence of other factors on the change in CIW was examined using linear regression for each heterogeneity estimator (Table 7). As an example of how to interpret the results in Table 7, the following text describes the results for the MD meta‐analyses using the HS estimator. Only the influence of *I* ^2^ was significant. The interpretation is different for *I* ^2^ than for the other parameters. For *I* ^2^ < 30%, the intercept is 0.0052. This means that when *I* ^2^ < 30%, a meta‐analysis performed with the HS estimator gives a 0.48% narrower CI than a meta‐analysis performed with the DL estimator (calculation: 0.9952 − 1 = −0.0048 = −0.48%). However, when the *I* ^2^ is greater than 30%, the difference is significantly greater; in this case, a meta‐analysis performed with the HS estimator gives a CI that is 18.24% narrower than a meta‐analysis performed with the DL estimator (calculation: 0.9952 − 0.1776 − 1 = − 0.1824 = − 18.24%).

**TABLE 7:** Other factors influencing the change in confidence interval width.

MD				
Parameter	Coefficient	Standard error	*t*	*p*
REML				
(Intercept)	0.8852	0.1064	8.3182	< 0.0001***
Number of studies	0.0340	0.0176	1.9377	0.0787
Unequality of number of patients	0.0003	0.0003	1.1107	0.2904
*I* ^2^ ≥ 30%	−0.0888	0.0994	−0.8933	0.3908
PM				
(Intercept)	0.7685	0.1322	5.8129	0.0001***
Number of studies	0.0425	0.0218	1.9478	0.0774
Unequality of number of patients	0.0003	0.0003	0.8892	0.3929
*I* ^2^ ≥ 30%	0.0262	0.1235	0.2118	0.8362
ML				
(Intercept)	0.8530	0.1217	7.0082	< 0.0001***
Number of studies	0.0276	0.0201	1.3714	0.1976
Unequality of number of patients	0.0001	0.0003	0.4830	0.6386
*I* ^2^ ≥ 30%	−0.1426	0.1137	−1.2537	0.236
HS				
(Intercept)	0.9952	0.0313	31.7621	< 0.0001***
Number of studies	−0.0030	0.0052	−0.5805	0.5733
Unequality of number of patients	0.0001	0.0001	0.9914	0.3428
*I* ^2^ ≥ 30%	−0.1776	0.0293	−6.0673	< 0.0001***
SJ				
(Intercept)	1.2554	0.2628	4.7777	0.0006***
Number of studies	0.0352	0.0434	0.8123	0.4339
Unequality of number of patients	0.0005	0.0007	0.8033	0.4388
*I* ^2^ ≥ 30%	−0.3686	0.2455	−1.5013	0.1614
HE				
(Intercept)	0.9273	0.3038	3.0523	0.0110*
Number of studies	0.0636	0.0501	1.2690	0.2306
Unequality of number of patients	−0.0008	0.0008	−0.9897	0.3436
*I* ^2^ ≥ 30%	−0.1399	0.2839	−0.4930	0.6317
EB				
(Intercept)	0.7685	0.1322	5.8129	0.0001***
Number of studies	0.0425	0.0218	1.9478	0.0774
Unequality of number of patients	0.0003	0.0003	0.8892	0.3929
*I* ^2^ ≥ 30%	0.0262	0.1235	0.2118	0.8362

OR				
Parameter	Coefficient	Standard error	*t*	*p*
REML				
(Intercept)	1.1741	0.1273	9.2248	< 0.0001***
Number of studies	−0.0074	0.0101	−0.7275	0.4774
Unequality of number of patients	−0.0000	0.0000	−0.4677	0.6463
*I* ^2^ ≥ 30%	0.0186	0.1211	0.1538	0.8797
PM				
(Intercept)	1.0148	0.1428	7.1064	< 0.0001***
Number of studies	−0.0070	0.0114	−0.6142	0.5477
Unequality of number of patients	0.0000	0.0000	1.1441	0.2694
*I* ^2^ ≥ 30%	0.2905	0.1359	2.1378	0.0483*
ML				
(Intercept)	0.9839	0.0896	10.9810	< 0.0001***
Number of studies	0.0038	0.0071	0.5255	0.6064
Unequality of number of patients	−0.0000	0.0000	−1.7460	0.1
*I* ^2^ ≥ 30%	−0.0689	0.0853	−0.8084	0.4307
HS				
(Intercept)	0.9466	0.0285	33.1721	< 0.0001***
Number of studies	0.0052	0.0023	2.2793	0.0367*
Unequality of number of patients	−0.0000	0.0000	−4.1342	0.0008***
*I* ^2^ ≥ 30%	−0.1694	0.0272	−6.2406	< 0.0001***
SJ				
(Intercept)	1.6391	0.5127	3.1968	0.0056**
Number of studies	−0.0196	0.0409	−0.4797	0.6379
Unequality of number of patients	0.0000	0.0000	2.0291	0.0594
*I* ^2^ ≥ 30%	0.3646	0.4879	0.7474	0.4657
HE				
(Intercept)	1.3854	0.3759	3.6857	0.0020**
Number of studies	−0.0254	0.0300	−0.8474	0.4092
Unequality of number of patients	−0.0000	0.0000	−0.9260	0.3682
*I* ^2^ ≥ 30%	0.1017	0.3577	0.2842	0.7799
EB				
(Intercept)	1.0149	0.1428	7.1077	< 0.0001***
Number of studies	−0.0070	0.0114	−0.6145	0.5475
Unequality of number of patients	0.0000	0.0000	1.1430	0.2699
*I* ^2^ ≥ 30%	0.2904	0.1359	2.1377	0.0483*

Abbreviations: EB: empirical Bayes estimator; HE: Hedges estimator; HS: Hunter–Schmidt estimator; MD: mean difference; ML: maximum‐likelihood estimator; OR: odds ratio; PM: Paule–Mandel estimator; REML: restricted maximum‐likelihood estimator; SJ: Hartung–Knapp–Sidik–Jonkman estimator.

* Significant results.

** Very significant results.

*** Highly significant results.

#### 3.3.6 Recalculation Using the HK Adjustment

The HK adjustment was generally not used in the meta‐analyses examined. The recalculation of the meta‐analyses with the HK adjustment showed that the differences between the eight different heterogeneity estimators were significantly smaller compared with the differences between the eight different heterogeneity estimators without the HK adjustment. The recalculation using the HK adjustment in terms of CIs and the number of significant results is shown in Tables 8 and 9.

**TABLE 8:** Recalculation of the RCIW and the number of significant results using HK adjustment.

Parameter	MD	OR
Ratio of CI width (RCIW), mean value (min.–max.)	REML: 1 (1–1.2)	REML: 1 (0.8–1.4)
	PM: 1 (1–1.1)	PM: 1.1 (0.9–1.8)
	ML: 1 (0.7–1.1)	ML: 1 (0.6–1.2)
	HS: 1 (0.9–1)	HS: 0.9 (0.8–1)
	SJ: 1.2 (1–1.8)	SJ: 1.4 (1–3.2)
	HE: 1 (0.7–1.6)	HE: 1.1 (0.5–2.9)
	EB: 1 (1–1.1)	EB: 1.1 (0.9–1.8)
Number of significant results, *n* (%)	DL: 3 (20%)	DL: 9 (45%)
	REML: 2 (13.3%)	REML: 9 (45%)
	PM: 3 (20%)	PM: 8 (40%)
	ML: 3 (20%)	ML: 9 (45%)
	HS: 3 (20%)	HS: 9 (45%)
	SJ: 2 (13.3%)	SJ: 8 (40%)
	HE: 2 (13.3%)	HE: 10 (50%)
	EB: 3 (20%)	EB: 8 (40%)

Abbreviations: DL: DerSimonian–Laird estimator; EB: empirical Bayes estimator; HE: Hedges estimator; HS: Hunter–Schmidt estimator; MD: mean difference; ML: maximum‐likelihood estimator; OR: odds ratio; PM: Paule–Mandel estimator; REML: restricted maximum‐likelihood estimator; SJ: Hartung–Knapp–Sidik–Jonkman estimator.

**TABLE 9:** Recalculation of the test for differences in the width of confidence intervals, using the HK adjustment.

Method	Coefficient	Standard error	*t*	*p*
MD				
EB	0.02	0.03	0.68	0.4961
HE	0.04	0.03	1.08	0.2849
HS	−0.02	0.03	−0.75	0.4557
ML	−0.02	0.03	−0.51	0.6106
PM	0.02	0.03	0.68	0.4961
REML	0.03	0.03	0.90	0.3707
SJ	0.16	0.03	4.89	< 0.0001***
OR				
EB	0.08	0.08	1.02	0.308
HE	0.07	0.08	0.97	0.3351
HS	−0.06	0.08	−0.75	0.4528
ML	−0.03	0.08	−0.34	0.738
PM	0.08	0.08	1.02	0.308
REML	0.04	0.08	0.52	0.6063
SJ	0.41	0.08	5.37	< 0.0001***

Abbreviations: EB: empirical Bayes estimator; HE: Hedges estimator; HS: Hunter–Schmidt estimator; MD: mean difference; ML: maximum‐likelihood estimator; OR: odds ratio; PM: Paule–Mandel estimator; REML: restricted maximum‐likelihood estimator; SJ: Hartung–Knapp–Sidik–Jonkman estimator.

*** Highly significant results.

## 4 Discussion

### 4.1 Key Findings and Methodological Concerns

This is the first study that attempts to qualitatively analyze the statistical methodology in meta‐analyses of THA. In the period 2022–2023, 24 meta‐analyses [19, 20, 21, 22, 23, 24, 25, 26, 27, 28, 29, 30, 31, 32, 33, 34, 35, 36, 37, 38, 39, 40, 41, 42] on THA were examined, of which 15 meta‐analyses reported MD outcome and 20 meta‐analyses reported OR outcome. As MD outcome, the HHS was extracted at the postoperative time point at which the parameter offered the most included primary studies. Similarly, the complication with the most included primary studies was extracted as OR outcome. The most important finding from the present study is that there are considerable differences in the significance of the meta‐analyses examined when different heterogeneity estimators are used. The DL and HS heterogeneity estimator in particular tended to produce false‐positive results. The DL estimator is widely used but has known limitations, particularly, in cases of high heterogeneity or small sample sizes. Its moment‐based approach tends to underestimate variance in such cases, leading to narrower CIs and inflated significance. Alternative estimators like REML and ML provide more stable variance estimates, reducing this risk. Prior studies have highlighted these limitations, reinforcing our findings [7, 64, 65, 66, 67]. Similarly, the HS estimator, which applies a correction for measurement error but does not fully account for study‐level variance, can also lead to inflated significance estimates. These effects are particularly pronounced when combined with the absence of HK adjustment, further amplifying statistical biases. Another very important finding is that the meta‐analyses examined generally did not use HK adjustment. Especially in combination with the weak DL heterogeneity estimator used in almost all cases, this leads to even more false‐positive results. Thus, the conclusions in some of the meta‐analyses examined have to be critically questioned. While our study primarily focuses on statistical methodology, we acknowledge the importance of assessing the influence of study quality on overall conclusions. Given the variability in AMSTAR 2 ratings, future research should investigate whether lower‐quality meta‐analyses disproportionately affect overall findings, as this could have implications for evidence‐based recommendations.

### 4.2 Impact of Heterogeneity Estimators on Effect Sizes and Confidence Intervals

In meta‐analysis, a heterogeneity estimator is a statistical measure used to assess the degree of variation or diversity between the results of individual included primary studies that are combined. It helps researchers determine whether the primary studies are sufficiently similar to be pooled, or whether there is significant heterogeneity that needs to be taken into account. Of the 24 meta‐analyses examined [19, 20, 21, 22, 23, 24, 25, 26, 27, 28, 29, 30, 31, 32, 33, 34, 35, 36, 37, 38, 39, 40, 41, 42], 23 [19, 20, 21, 22, 23, 24, 25, 26, 27, 28, 29, 30, 31, 32, 33, 34, 35, 37, 38, 39, 40, 41, 42] used the DL heterogeneity estimator and only one [36] used the PM heterogeneity estimator. However, previous research has raised some concerns about the DL heterogeneity estimator, as it led to a high rate of false positives [7, 64, 65, 66, 67]. To estimate the magnitude of the differences when using the different heterogeneity estimators, each meta‐analysis examined was recalculated for each of the eight heterogeneity estimators. The calculation of the RES with the different heterogeneity estimators showed that the effect size did not differ significantly in OR meta‐analyses with a RES value around 1. However, in MD meta‐analyses, the effect sizes for the different heterogeneity estimators showed significant differences, as the RES value ranged from 0.2 to 3.7 in individual cases. This means that in some of the meta‐analyses examined, the effect size obtained was 0.2 to 3.7 times larger than that obtained using DL. When these findings are applied to orthopedic practice, their relevance becomes even clearer. It makes a huge difference whether the effect size of HHS is 10 points or only 2 points with a RES value of 0.2. The relevance becomes even clearer considering that this difference in HHS is a minimally clinically important difference (MCID) [68]. A 95% CI means that there is a 5% chance that the results are wrong. The larger the CIW, the less accurate the estimate. Again, there were relevant differences in CIW in some MD meta‐analyses and in some OR meta‐analyses. The number of significant results showed how many of the 15 MD and 20 OR outcome parameters led to significant results when the meta‐analyses examined were recalculated using the different heterogeneity estimators. The number of significant results ranged from 2 to 5 (13.3%–33.33%) out of 15 for MD outcome parameters and from 7 to 9 (35%–45%) out of 20 for OR outcome parameters. This finding again strongly questions the reliability of the conclusion of some meta‐analyses, considering that the use of a more reliable heterogeneity estimator may change an important THA outcome such as HHS or any type of THA complication from showing a significant difference to showing a nonsignificant difference or vice versa.

Our study examined eight heterogeneity estimators commonly used in meta‐analyses, selected based on their methodological relevance and prior research on their performance. The choice of estimator significantly influenced effect sizes, CIs, and statistical significance. Given the widespread use of the DerSimonian–Laird estimator despite its limitations, we highlight the need for more robust alternatives such as REML or Sidik–Jonkman, particularly, when heterogeneity is high. Our findings reinforce the importance of carefully selecting heterogeneity estimators and applying the HK adjustment to improve result reliability.

### 4.3 HK Adjustment and Implications for Clinical Practice

Another advantage of the present study is that it investigated the influence of the HK adjustment on the results of the meta‐analyses examined [14]. The HK adjustment is a statistical method used to deal with the issue of heterogeneity in meta‐analyses. It adjusts the standard errors of effect sizes to provide more accurate CIs and hypothesis tests when there is variability in study results. The HK adjustment was generally not used in the meta‐analyses examined, which is considered to be a shortcoming. However, the usage of the HK adjustment is advisable when calculating CIs. After recalculating the meta‐analyses examined using the HK adjustment, the differences between the heterogeneity estimators became much smaller compared with the differences between the heterogeneity estimators without HK adjustment. In particular, the DL and HS heterogeneity estimators no longer seemed to produce false‐positive results.

Our findings highlight the risk of misleading conclusions in meta‐analyses of THA due to inappropriate heterogeneity estimators, particularly, the widespread use of the DerSimonian‐Laird method without HK adjustment. This can distort clinical decision‐making by overstating or understating the benefits and risks of specific surgical techniques or implants. To ensure reliable evidence, we recommend adopting more robust heterogeneity estimators and standardizing statistical methods in meta‐analyses. Incorporating these improvements into future guidelines could enhance the validity of meta‐analytic evidence, ultimately leading to better‐informed clinical recommendations and improved patient outcomes. The key recommendations are summarized in a fact box (Table 10) for clarity and practical guidance.

**TABLE 10:** Fact box: Recommendations for future meta‐analyses.

Category	Key points
Key findings	− Standard heterogeneity estimators (DL, HS) tend to produce false‐positive results. − HK adjustment significantly reduces differences between estimation methods and improves result reliability. − Many meta‐analyses lack robust methods for uncertainty assessment, leading to potential bias in conclusions.
Recommendations	✔ Use more reliable heterogeneity estimators such as REML, PM, or ML instead of DL. ✔ Always apply Hartung–Knapp adjustment to minimize bias and improve robustness. ✔ Ensure a clear assessment of heterogeneity and avoid weak estimators that may inflate significance.

Abbreviations: DL: DerSimonian–Laird estimator; HK: Hartung–Knapp; HS: Hunter–Schmidt estimator; ML: maximum‐likelihood estimator; PM: Paule–Mandel estimator; REML: restricted maximum‐likelihood estimator.

### 4.4 Strengths and Limitations of the Study

The present study has several strengths and limitations. It is the first study to attempt such a comprehensive qualitative analysis of the statistical methodology in meta‐analyses of THA. It involved substantial statistical effort and required high‐quality statistical skills to recalculate 15 MD and 20 OR meta‐analyses for eight different heterogeneity estimators. In addition, the entire work was recalculated with the HK adjustment. A limitation of this study is the restriction to meta‐analyses published in PubMed between 2022 and 2023. This selection was made to ensure a manageable yet representative sample, as our study involved extensive recalculations of statistical methods, which would not have been feasible for a larger dataset. While this approach allowed for a systematic evaluation of statistical methodologies, it may limit the generalizability of our findings to older meta‐analyses or those indexed in other databases. The differences in prediction intervals using different heterogeneity estimators were not assessed.

## 5 Conclusion

Without the HK adjustment, the results depend strongly on the chosen heterogeneity estimator and there is a risk of false‐positive results, especially for the widely used DL heterogeneity estimator. With HK adjustment, the choice of heterogeneity estimator seems to play a less important role. We recommend the use of more reliable heterogeneity estimators as well as the HK adjustment as a measure to improve the statistical methodology of meta‐analyses.

## Author Contributions

**Nikolai Ramadanov:** conceptualization, investigation, writing – original draft, methodology, validation, visualization, writing – review and editing, software, formal analysis, project administration, data curation, supervision, resources. **Maximilian Voss:** data curation, investigation, software, formal analysis. **Radharani Michelle Diallo:** software, formal analysis, data curation, investigation. **Jonathan Lettner:** software, formal analysis, data curation, investigation. **Hassan Tarek Hakam:** writing – review and editing. **Robert Prill:** writing – review and editing. **Roland Becker:** writing – review and editing. **Robert Hable:** conceptualization, investigation, writing – review and editing, validation, methodology, software, formal analysis, project administration, supervision.

## Conflicts of Interest

The authors declare no conflicts of interest.
